# Exosomes in schizophrenia: Pathophysiological mechanisms, biomarkers, and therapeutic targets

**DOI:** 10.1192/j.eurpsy.2022.2319

**Published:** 2022-09-09

**Authors:** Yu Wang, Nousayhah Amdanee, Xiangrong Zhang

**Affiliations:** 1 Department of Geriatric Psychiatry, The Affiliated Brain Hospital of Nanjing Medical University, Nanjing, Jiangsu, China; 2 Department of Psychiatry, The Affiliated Xuzhou Oriental Hospital of Xuzhou Medical University, Xuzhou, Jiangsu, 221004, China

**Keywords:** Biomarkers, exosomes, miRNAs, schizophrenia, therapeutic targets

## Abstract

While schizophrenia (SCZ) is a devastating psychiatric disorder that detrimentally affects a significant portion of the worldwide population, its diagnosis is traditionally based on a relatively subjective assessment of current symptoms and medical history, devoid of an objective diagnostic modality. Antipsychotic medications are commonly used in the treatment of SCZ; however, some patients have low remission rates or forsake treatment due to the associated multiple side effects, resulting in recurrent episodes of the disease and poor prognosis. These situations imply that the diagnosis, treatment, and prognosis of SCZ need to be improved to increase the odds of a better outcome. Mounting studies have found that extracellular vesicles (EVs) play essential roles in the central nervous system. They are implicated in several mechanisms closely associated with SCZ such as cellular communication and synaptic plasticity. They can additionally exhibit neuroprotective and therapeutic effects. Since they possess distinct constituents, are readily available, easily detectable, and dependent on the internal environment, they can potentially serve as reliable biomarkers for disease diagnosis. Moreover, their biological configuration along with their ability to increase the bioavailability of their constituents and modulate intricate intracellular reactions in target cells, propel EVs as new targets for treatment. This review paper summarizes relevant research pertaining to the roles of EVs in SCZ, with the aim of improving insights into SCZ pathogenesis and evaluating EVs as potential biomarkers in the diagnosis and treatment of SCZ.

## Introduction

Schizophrenia (SCZ) is currently one of the most debilitating diseases around the world. SCZ patients with comorbidities have a high degree of disability [1] that imposes a heavy financial burden on the affected individual, their family members, and the society.

With a heritability of about 80% [2], SCZ mostly occurs in late adolescence and early adulthood [3] with positive (hallucinations, delusions, etc.,) and negative (apathy, cognitive symptoms, etc.,) symptoms [4]. While SCZ patients suffer from an average of 14.5 years of potential life loss, early diagnosis and interventions might improve their condition [5]. At present, the diagnosis of SCZ includes a relatively subjective assessment of current symptoms and history, including various clinical interview-based tools, but lacks objective diagnostic biomarkers [6]. Studies have shown that SCZ has a high rate of misdiagnosis. In particular, psychotic bipolar disorder continues to be frequently misdiagnosed as SCZ or other psychotic illnesses. Approximately 30% of bipolar disorder patients are initially misdiagnosed with SCZ, thereby delaying the treatment and prognosis of the disease [7].

Since antipsychotic medications, the first-line treatment for SCZ, have a long-documented history of undesirable metabolic side effects [8], roughly 60% of patients abandon their treatment due to these side effects, which include metabolic syndrome [9, 10]. In addition, there are about 40% of patients who have poor treatment response, named refractory SCZ [11]. Moreover, antipsychotic drugs mainly improve the positive symptoms of SCZ, and are thus ineffective for patients who predominantly exhibit negative and cognitive deficits [12] which are closely related to prognosis and the likelihood of integrating back into society. As such, SCZ requires new diagnostic and therapeutic modalities so as to ameliorate psychotic symptoms and prognosis. The application of reliable biomarkers could potentially remediate these issues in clinical practice.

Extracellular vesicles (EVs) are currently a hot research topic in diverse scientific fields [13]. They were once merely considered as disposal systems of cellular waste, but have recently emerged as a novel element capable of mediating intracellular communication via an exchange of biomolecules [14]. Exosomes are bilayer membrane-enclosed EVs that are 30–150 nm in size and with varying compositions. In vivo, they can be found abundantly in various types of body fluids, such as blood, urine, cerebrospinal fluid, amniotic fluid, and ascites [15–18]. Diverse exosomes also differ in their components, including distinct protein, lipid, and nucleic acid profiles [19]. The presence of CD9, CD63, and CD81 in EVs has previously been established as exosome markers. In addition, the biogenesis and subsequent release of exosomes are fairly dependent on the physiological state as well as the conditions of the originating cells. Since they have distinct constituents, are readily available, easily detectable, and dependent on the internal environment, exosomes can potentially serve as biomarkers for the evaluation and diagnosis of diseases. It was revealed that exosomes isolated from blood or urine were effective diagnostic and prognostic markers in cancer [20] and CNS disease [21].

Since exosomes have the ability of crossing the blood–brain barrier [22], their implication in neuropsychiatric diseases is not surprising. Exosomes promote or limit the aggregation of unfolded and abnormally folded proteins in the brain [23–26], thereby inducing neurodegenerative diseases. Moreover, exosomes are involved in several physiological mechanisms closely related to SCZ, including cell communication [27], regulation of synaptic plasticity, and nerve regeneration processes [28]. On the other hand, exosomes can exert neuroprotective effects, such as disrupting the development of neurotoxic oligomers [29] or removing them from cells [30], thus indicating the ability of exosomes in negating the development of diseases. In vivo, targeted drug delivery is a promising treatment modality for many refractory diseases. Specifically, exosomes secreted by mesenchymal stem cells are potential drug carriers owing to their physiological characteristics [31]. It was shown that the injection of mesenchymal stem cell-derived exosomes into mouse models resulted in the direct transport of exosomes to damaged brain areas in contrast to an absence of this phenomenon in healthy controls (HCs) [32], suggesting that exosomes could potentially treat central nervous system lesions.

**Figure 1.**
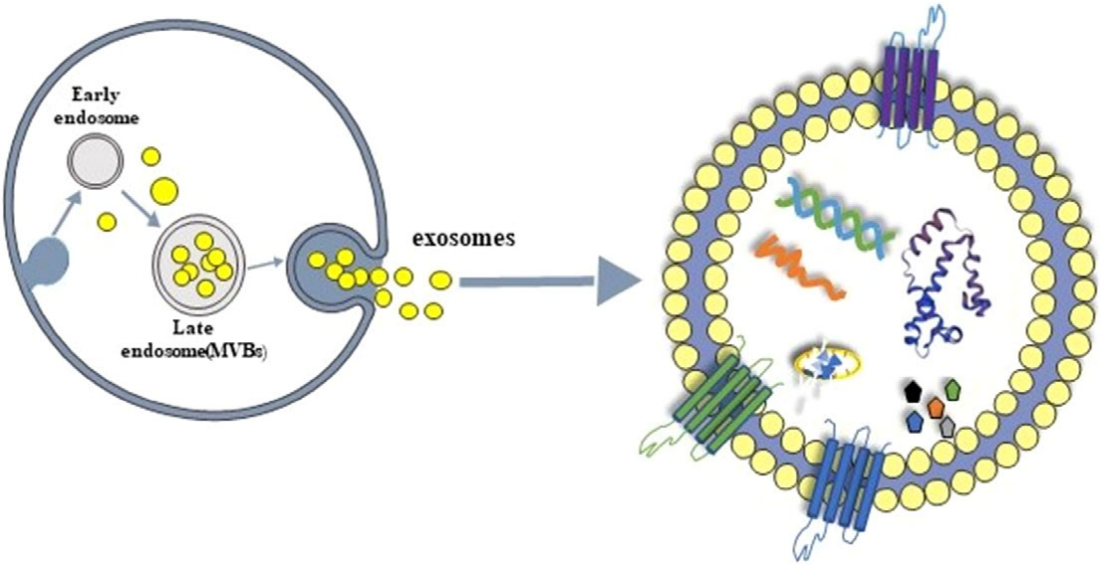
Exosome production and typical morphology.

Based on the aforementioned findings, exosomes have the potential to play a central role in the evaluation, diagnosis, and treatment of neuropsychiatric diseases, in particular, SCZ. In this line, the present review summarizes the current research pertaining to exosomes in SCZ, including their functional role in SCZ pathology and prognosis in addition to their potential value in therapeutic applications. We also discuss existing information gaps and avenues of expanding prospective areas of investigation that would help propel exosomes as biomarkers, in order to gain further insight and translational utility (Figure 1).

## Exosomes in the Pathogenesis and Diagnosis of Schizophrenia

### Exosomal noncoding RNA

#### Exosomal microRNAs

Exosomes transmit a variety of signaling molecules, such as proteins, mRNA, and noncoding RNA that most notably include microRNAs (miRNA) [33]. MiRNAs are considered as key functional elements of exosomes which could influence cells via the negative regulation of gene expression [34] and interaction with cell receptors as ligands [35]. These processes along with the involvement of miRNA in the regulation of nearly every cellular pathway, portray miRNAs as highly promising biomarkers for disease progression and treatment response, especially in neurodegenerative diseases [36–38].

Since the constituents of MiRNAs are rapidly redistributed and altered when cultured human neurons are depolarized, the predicted targets of regulated miRNA are functionally clustered, revealing enrichment of genes involved in plasticity-related processes including synaptic activity, protein localization and neuron morphogenesis. The functionality of predicted target genes was supported by the observed genome-wide changes in gene expression, suggesting that miRNAs are important regulatory components in the dynamics of normal synaptic function. As synaptic function is thought to be compromised in neurodegenerative and neuropsychiatric conditions, it is plausible that these molecules and their role in translational homeostasis are disrupted in SCZ [39, 40]. Goldie et al. [39] demonstrated that in response to depolarization, there was a downregulation of miRNAs in neurons accompanied by the simultaneous release of MAP1b-containing exosomes that are usually associated with synaptic plasticity. These findings indicate that miRNA release and function might be impaired in neuropsychiatric disorders since synaptic dysfunction typically characterizes neuropsychiatric disorders. In a post-mortem brain study, multiple statistical analyses of microarray data were performed and revealed that certain exosomal miRNAs were differentially expressed in the prefrontal cortices of SCZ patients in comparison to controls, and the top 21 of these differentially expressed miRNAs could differentiate SCZ from HCs. In particular, RT-PCR validation confirmed that miR-497 in SCZ had a significantly higher expression when compared to controls [41]. Zhai et al. [42] revealed through luciferase activity assay that fibroblast growth factor 2 (FGF2) are a direct target of miR-497 and are regulated by miR-497 in microglia. The authors also demonstrated that the knockdown expression of miR-497 inhibited the activation of microglia and production of proinflammatory cytokines including IL-6, IL-1β, MCP-1 and TNF-α in CUS-induced rats while an overexpression of FGF2 inhibited miR-497-induced proinflammatory cytokines and iNOS expression. These findings are consistent with the point that FGF2 reduces the level of pro-inflammatory factors and improves behavior in neurodegenerative diseases [43]. Therefore, further studies on the role of miR-497 and its targets in SCZ can help to broaden the current knowledge on the pathogenesis of SCZ and additionally develop targeted drugs.

Astrocytes contain abundant miR-223 that are secreted through exosomes [44]. A large body of literature has divulged that miR-223 can participate in cellular communication via exosomes [45]. miR-223 was also shown to target glutamate receptors. It was documented that an overexpression of miR-223 led to chronic hypofunction of the *N*-methyl-d-aspartate receptor (NMDAR) and lower synaptic activity [46]. Amoah et al. [44] revealed that miR-233 was increased at the mature miRNA level in the orbitofrontal cortex (OFC) of SCZ patients. The authors also reported that miR-223 was enriched within astrocytes and was highly expressed in glial and neuronal exosomes. They additionally demonstrated that the addition of astrocytic exosomes in cortical neurons resulted in an increased neuronal miR-223 expression and reductions in Grin2b mRNA levels, which were rescued following inhibition of miR-223 in the astrocytes. Furthermore, using a 2-day treatment with olanzapine and haloperidol, the expression of miR-223 was significantly reduced in both neuronal pellets and neuronal exosomal fractions, suggesting a reduction in miR-223 synthesis within neurons. However, treatment with the same antipsychotics reduced miR-223 expression in astrocytic pellets but increased miR-223 levels in the exosomal fraction, indicating that miR-223 is enriched in the astrocytes and is secreted via exosomes while antipsychotics reduce miR-223 synthesis in neurons but increase miR-223 exosomal secretion in the astrocytes of the brain parenchyma. Taken together, the results showed that a psychosis-altered and glial-enriched miRNA, whose expression could be regulated by antipsychotics, is secreted by exosomes in order to inhibit neuronal NMDA receptor gene expression.

In addition to their key role in the pathogenesis of SCZ, blood-derived exosomal miRNAs have shown great potential as objective biomarkers for the diagnosis of various diseases, such as cancer [47], AD [48], PD [49], and so on. Du et al. [50] conducted a genome-wide analysis of exosomal miRNAs in the first episode SCZ and discovered that 353 miRNAs were differentially expressed, among which levels of hsa-miR-206, hsa-miR145-5p, hsa-miR-133a-3p, hsa-miR-144-5p, hsa-miR144-3p, and hsa-miR-184 were two times higher in SCZ patients compared to HCs. They additionally clarified that 11 miRNAs from these differentially expressed miRNAs distinguished between SCZ and HC groups with an accuracy of approximately 90% in training samples and 75% in testing samples. Altogether, these studies confirmed the crucial involvement of exosomal miRNA in SCZ which could serve as potential diagnostic biomarkers. Hence, in-depth studies on the specific mechanism of differentially expressed miRNA in exosomes are necessitated.

A previous study established that transplanting exosomes from SCZ patients into mice through a tail vein injection (8 mg/kg) induced aberrant behaviors similar to those in the animal model of SCZ, such as pre-pulse inhibition and altered social preferences compared to saline-treated and HCs blood exosome-recipient mice. Although these symptoms lack specificity, behavioral tests (open-field, prepulse inhibition, social interaction tests, elevated plus maze, novel object recognition tests) were significantly different [51]. In these mice with transplanted SCZ exosomes, Du et al. [51] detected differentially expressed mRNA (999 mRNAs were down-regulated and 888 were up-regulated) and significantly enriched pathways involved in synaptic transmission, neurodevelopment, and behavior, which have been strongly associated with SCZ. They also pointed out that these functionally connected DEGs, as analyzed by protein–protein interaction networks, included glutamate receptors (Grin1, Grin3a, and Grik5), DRD2, BDNF, and Camk2a, which are largely associated with SCZ pathogenesis. Moreover, the differentially expressed exosomal miRNAs in SCZ were predicted to regulate a considerable amount of differentially expressed genes in the prefrontal cortex and hippocampus of mice with transplanted SCZ blood exosomes. These results are in agreement with the theories that exosomal miRNA dysregulations might contribute to the onset and/or development of SCZ.

In another study, Du et al. [52] investigated the metabolomics of exosomes with UPLC-MS/MS and identified 25 metabolites that were significantly and differentially expressed in SCZ patients. These metabolites were not influenced by drug treatment and could effectively distinguish SCZ from the control group. Their findings indicated that the significantly and differentially expressed metabolites were related to phenylalanine, tyrosine, tryptophan and glycerol phospholipid metabolic pathways, thus supporting the membrane phospholipid dysfunction hypothesis in SCZ. However, this research did not consider potential confounding factors, such as smoking status and socioeconomic status, which might have influenced the concentration of blood exosomal metabolites. The combination of miRNAs and metabolites from blood exosomes could possibly improve the diagnostic accuracy for SCZ. Therefore, more studies are needed to translate the findings of blood exosomal metabolite biomarkers into benefits for patients with SCZ.

Furthermore, antipsychotic drugs can also affect miRNA expression levels in exosomes. In blood-derived exosomes of 11 first-episode drug-naïve patients with SCZ, miR-203a-3p levels were increased and its target- the antioxidant DJ-1 protein demonstrated decreased compared with healthy controls. Surprisingly, after 6 weeks of olanzapine monotherapy (The dose of olanzapine was either 15 mg *n* = 5 or 20 mg *n* = 6), the expression level of DJ-1 was increased and miR203a-3p was decreased. Thus, olanzapine may exert a therapeutic effect by reversing the expression levels of miR203a-3p and DJ-1 in exosomes [53].

#### Exosomal circular RNA

Advancement in RNA sequencing techniques has allowed better-quality investigation of circular RNA (circRNA). The expression of circRNAs is enriched, well conserved among species, highly cell‐type specific, and relatively more stable than linear RNAs. CircRNAs also have an impact on the levels of gene expression by functioning as miRNAs sponges and play a crucial role in gene regulation at the transcriptional or post‐transcriptional levels. In addition, they can function as protein scaffolds or sequester proteins and thus participate in physiological processes [54]. CircRNAs is found abundantly in the brain and can be loaded into exosomes to cross the blood–brain barrier. CircRNAs are mainly concentrated on synapses, which is related to synaptic plasticity [55]. Accordingly, circRNA could be a potential reliable diagnostic biomarker of SCZ. It was discovered that circRNA was aberrant in the dorsolateral prefrontal cortex of SCZ patients, and deficits in circHomer1a, a neuronal-enriched circRNA, in the dorsolateral prefrontal cortex were positively correlated with the age of SCZ onset [56]. In mice, a knockdown of circHomer1a in mouse OFC led to specific behavioral deficits in reversal learning, indicating the possibility that dysregulation of this circRNA in the brain of subjects with SCZ could be associated with some of the cognitive disturbances observed in these psychiatric disorders [57].

A recent study documented that circRNAs in plasma exosomes were differentially expressed in SCZ [58]. In this study, Tan G et al. identified alterations in 44 exosomal circRNAs, comprising 38 up-regulated and six down-regulated circRNAs by high‐throughput sequencing. Through qRT-PCR, they also validated the presence of four differentially expressed circRNAs that contained binding sites of numerous miRNAs, including miR-34a and miR-499a which were involved in SCZ etiology. Furthermore, bioinformatics analysis pointed out that the differentially expressed circRNA was implicated in the phosphorylation of certain proteins, thus participating in the signaling cascade conduction of multiple protein synthesis which might be associated with the abnormality of multiple proteins in SCZ. Various circRNAs were also enriched in specific signaling pathways, such as MAPK and Notch pathways, that play essential roles in adult neurogenesis [59]. These results provide a new window for understanding the pathogenesis of SZ at molecular levels. However, circRNA is still under-researched in SCZ and requires more attention.

#### Exosomal long non-coding RNA

The contribution of long non-coding RNA (lncRNAs) to the pathogenesis of SCZ has also been documented. Since the abnormally expressed lncRNAs in the brain can be transported via exosomes to cross the blood–brain barrier into peripheral blood and can accurately reflect the pathological state of brain disease, lncRNA are thus of great clinical value [60]. Guo et al. [61] selected 15 lncRNAs associated with SCZ in order to analyze the expression levels of serum exosomes in 152 subjects by RT-qPCR and evaluate their diagnostic values. The researchers corroborated that the expression of MIAT and PVT1 in serum exosomes of unmedicated SCZ patients was significantly different relative to HCs. ROC analysis revealed that MIAT in serum exosomes had the best discriminatory performance between SCZ and HCs. The authors also demonstrated that risperidone could affect the expression of MIAT and PVT1, indicating the close association of lncRNAs to the onset and progression of SCZ [61]. However, only one study reported the association between exosomal lncRNAs and SCZ. Moreover, the authors did not describe the EV extraction method and thus, it is possible that the lncRNAs that were picked up in this study are contaminants and not actually encapsulated in exosomes.

### Exosomal mitochondrial dysfunction

There is a growing number of reports that have confirmed the existence of oxidative stress and mitochondrial dysfunction in SCZ [62, 63]. It was previously reported that the enzymatic activity of complex V (ATP synthase) of the electron transport chain was deficient in astrocyte-derived extracellular vesicles or exosomes (ADEVs, immunoblotting with ACSA-1 biotinylated antibody) of 10 first episode SCZ patients but not in HCs. In contrast to HCs, the patient group exhibited significantly lower ADEV levels of the membrane-associated signaling proteins, MFN2 and CYPD, that typically integrate and regulate numerous mitochondrial functions. Most distinctively in SCZ patients, there was also a substantial reduction in the ADEV and neuron-derived extracellular vesicles or exosomes (NDEVs, immunoblotting with L1CAM biotinylated antibody) levels of the short open-reading frame encoded peptides, humanin, and MOTS-c, which are known to improve neuronal survival and regulate aspects of metabolism, respectively [64].

Mitochondrial metabolic disorders were associated with social behavioral impairment via mitochondrial sequestration of gamma-aminobutyric acid (GABA) and lower GABAergic signaling [65]. Parvalbumin (PV) interneuron is the predominant GABAergic neuron and the principal cause of excitatory pyramidal neurons perisomal inhibition, which is abnormal in the cerebral cortex of SCZ patients [66]. In the prefrontal PVIs of redox dysregulated mice (Gclm-KO + GBR), researchers found that an overexpression of miR-137, promoted by oxidative stress, yielded a reduction of COX6A2 in PV interneurons and an accumulation of deficient mitochondria accompanied by low levels of mitophagy pointers that additionally aggravated oxidative stress and undermined PV interneurons. After Gclm-KO + GBR mice were treated with MitoQ (a selective mitochondria-targeted antioxidant), the abnormalities in cell body counts and staining intensity of PVI processes in the ACC were restored to wild-type levels, and increased miR-137 plasmatic levels were also reversed, suggesting that MitoQ can reverse OxS-induced miR-137 overexpression in addition to decreasing COX6A2 levels, mitophagy, and PVI impairment, and thus highlighting that the upregulation of miR-137 and subsequent mitophagy defects constitute one of the molecular mechanisms underlying the OxS-induced PVI impairment [67]. When translating this study onto human subjects, there were higher plasma exosomal miR-137, lower COX6A2, and altered mitochondrial autophagosomes in SCZ patients. Those with mitochondrial impairments displayed more severe clinical symptoms and cognitive impairments. Additionally, higher miR-137 levels and lower levels of COX6A2 were linked with poorer cognitive functions in these patients. Taken together, these findings suggested that exosomal miR-137 and COX6A2 might collaboratively distinguish whether PV interneuron-mediated mitochondrial damage is present in the early stages of SCZ, and could thus serve as potential treatment targets and predict the occurrence of the disease in advance.

### Exosomal protein abnormalities

Cognitive deficits are widespread in SCZ and remain a major predictor of functional impairment. The analysis of amyloid in SCZ patients with cognitive impairments revealed that in contrast to HCs, there was a higher level of astrocyte-derived exosomal (ADE) amyloid-beta 1–42 which was able to sensitively and specifically discriminate patients from HCs. Furthermore, an increase in ADE phosphorylated T181 tau concentrations was related to a lower level of negative symptoms and higher oxidative stress [68]. These findings point out that exosomal Aβ and tau might mediate cognitive deficits in SCZ. Hence, exosomal-derived proteins might have future diagnostic and therapeutic implications. However, since this study was cross-sectional and causality cannot be inferred, longitudinal investigations of the relationship between exosomal Aβ and cognition are warranted. In addition, the function of plasma levels of exosomal Aβ and tau along with their association with CSF protein levels have not been fully elucidated. These markers might provide an innovative and relatively non-invasive approach to recognizing the role of Aβ and tau in SCZ.

Dystrobrevin Binding Protein 1 (DTNBP1) is a risk gene for SCZ [69] that encodes dysbindin. DTNBP1 serves as a synaptic protein that forms multiple protein complexes and is involved in regulating the transport of neurotransmitters and receptors [70]. The toxic aggregates of dysbindin-1B can have a great impact on the viability of neurons. Aggregates of dysbindin-1B can be encapsulated by exosomes and propagate between neurons both in vitro and in vivo. Through exosome-mediated propagation, the deposits of dysbindin-1B exert toxic effects on recipient neurons located a long distance away from the initial aggregation site in mice brains. The exosome-mediated propagation and neurotoxicity in affected neuronal circuitry, resulting from aggregated dysbindin-1B, potentially imply that the accumulation of certain proteins in SCZ might cause a greater impairment to neurons and might be associated with the deterioration of cognitive function in SCZ patients [71]. However, more studies are still needed to clarify the association of aggregated dysbindin-1B with cognitive impairment in SCZ.

Since exosomal proteins might play a role in SCZ, researchers examined neuropathology-relevant protein biomarkers, including synaptophysin which is a-II-Spectrin and Glial Fibrillary Acid Protein (GFAP), in the circulating plasma-derived exosomes of chronic SCZ and HCs [72]. The results depicted that the concentration of a-II-Spectrin was lower in SCZ while the exosomal samples from both groups had a similar concentration of synaptophysin, implying the presence of neuronally derived exosomes (NDE) irrespective of disease status. Since a-II-Spectrin is not only a neuronal/synaptic cytoskeletal protein but is also an integral constituent of EVs, whether the reduced a-II-Spectrin levels in SCZ reflect an alteration in exosome production or secretion related to the illness, remains unclear and necessitates further investigation. Additionally, the authors did not specifically extract NDE from the plasma samples. Thus, future studies should specifically examine the expression of synaptophysin and a-II-Spectrin in NDE in SCZ.

Elevated GFAP expression is associated with increased astrocytic activation and inflammation in the CNS. Although a study reported no difference in free (non-exosomal) GFAP in CSF and serum samples from first-episode SCZ and controls [73], it was disclosed that there was a higher concentration of GFAP in the plasma exosomes of SCZ participants. Given the cell-of-origin specificity of exosomes and the enrichment and stability of their cargo protein, analyzing exosomal cargos might provide a better possibility for identifying neuropathology-relevant proteins. Further studies are needed to determine if distinct patterns of exosomal GFAP expression are detectable in subsets of SCZ and to analyze the effect of antipsychotic medication exposure on exosomal protein expression.

### Exosomal abnormal insulin signaling pathway

Autopsy studies of SCZ subjects have observed a reduction in the expression of insulin receptors in the frontal cortex and lower downstream signaling proteins, such as protein kinase B (AKT), glycogen synthase kinase 3 beta (GSK3β), and the mammalian target of rapamycin (mTOR) phosphorylation [74–76]. 70 kDa ribosomal protein S6 kinase (P70S6K), a vital enzyme in the insulin signaling pathway, was associated with poorer language learning and brain glucose in SCZ, inferring that SCZ patients might exhibit impaired insulin sensitivity and insulin resistance in the brain which was associated with learning and memory dysfunctions [77]. Magnetic resonance spectroscopy analysis of neuronal-derived extracellular vesicles found that neuronal insulin signaling abnormalities were already present in the first episode of SCZ, and were linked to memory impairment and lower brain glucose utilization in the occipital cortex [78]. Insulin disturbances might also be implicated in the development of SCZ by influencing the normal functioning of neurotransmitters or the immune system [79]. Nonetheless, the precise mechanism of the aberrant insulin signaling system in SCZ is currently unknown.

## Exosomes in the Potential Treatment of Schizophrenia

Numerous studies have described the potential role of EVs in the treatment of various diseases, including cancer [80], cardiovascular diseases [81], and most importantly, neurodegenerative diseases [82, 83]. Since the information within EVs can modify the biological reactions in recipient cells, EVs-mediated activities can result in the propagation or suppression of disease progression. Subsequently, they have the potential to be utilized to distribute various therapeutic loads to targeted cells through the modulation of intricate intracellular processes. They additionally have the advantages of increasing the bioavailability of their constituents and reducing side effects owing to their biological configuration [22]. However, since the existing research is performed a cellular level or using animal models, there is still a long way to go before EVs can be used in clinical treatment.

Mesenchymal stem cells (MSCs) are a kind of adult multipotent cells having self-renew capacity and differentiation potential. The primary mechanism of MSCs mediated regeneration effect is a secretome-based paracrine activity. MSCs-derived exosomes (MSC-exos) are one of the first bioactive factors which were isolated and characterized from human ESC-derived MSCs conditioned medium [84]. Compared to other cell types, MSCs are the most prolific exosome producer that can be isolated by surface markers such as CD73, CD44, and CD29 [85]. Exosomes secreted by MSCs are the most appropriate to transport therapeutic loads to target cells due to their favorable features which include small size, low immunogenicity, long half-life, decent permeability, and good biocompatibility [86]. MSC-exos, which were isolated from hMSC derived from bone marrow aspirates of normal healthy donors, diminished neuroinflammation and promoted neurogenesis as well as angiogenesis in animal models of epileptic [87]. Moreover, in a rat model, MSC-exos improved spatial learning disability and enhanced functional recovery of traumatic brain injury [88]. Researchers have demonstrated that miRNA mimic can be packaged into exosomes secreted by MSCs which can serve as an alternative material for the treatment of glioma due to their key role in delivering therapeutic drugs or genes that have antitumor effects [89], thereby suggesting that MSCs not only secrete a large number of exosomes that participate in genetic communication but also deliver the exogenous miRNA mimics which provide an efficient route of therapeutic miRNA delivery.

In a recent study, researchers combined classical X-ray computed tomography (CT) with gold nanoparticles as labeling agents to monitor intranasally administrated bone marrow MSC-exos in different brain pathologies, comprising stroke, autism, Parkinson’s disease, and Alzheimer’s disease. The MSC-exos explicitly targeted and accumulated only in pathologically relevant brain regions up to 96 h after administration while they exhibited a diffuse migration pattern and were progressively cleared by 24 h in healthy brains. There was a selective uptake of MSC-exos only by neuronal cells and not by glial cells in pathological brain regions. Moreover, the Alzheimer’s and autism mice models were intranasally treated with PKH26-labeled MSC-exos (2.8 × 109 exosomes, total volume of 20 μL) to examine whether neuroinflammation in pathological brains was associated with specific MSC-exo migration. The frontal cortex, striatum, hippocampus, and cerebellum were immunostained by the marker of activated microglia CD11b after 96 h whereby immunostained cells were correlated with the PKH26 signal. The results showed that the anti-CD11b and PKH26 fluorescent signal intensity was low and uncorrelated in all the examined regions in healthy mice while a high CD11b signal was observed especially in the hippocampus and striatum of the Alzheimer’s mice model, and a high CD11b signal was found in the cerebellum of the autism mice model. In both models, there was a significant correlation between CD11B and PKH26 signal intensities, thus demonstrating a strong association between MSC-exos and neuroinflammatory signals in pathological brains. These findings indicate that the pathology-specific homing patterns of MSC-exos were driven by inflammation and depict the key role of MSC-exos in the pathological brain. Therefore, MSC-exos could potentially be employed for diagnostic and therapeutic purposes [32]. Further research in SCZ should focus on the development of a treatment that target pathological brain regions [32].

**Figure 2.**
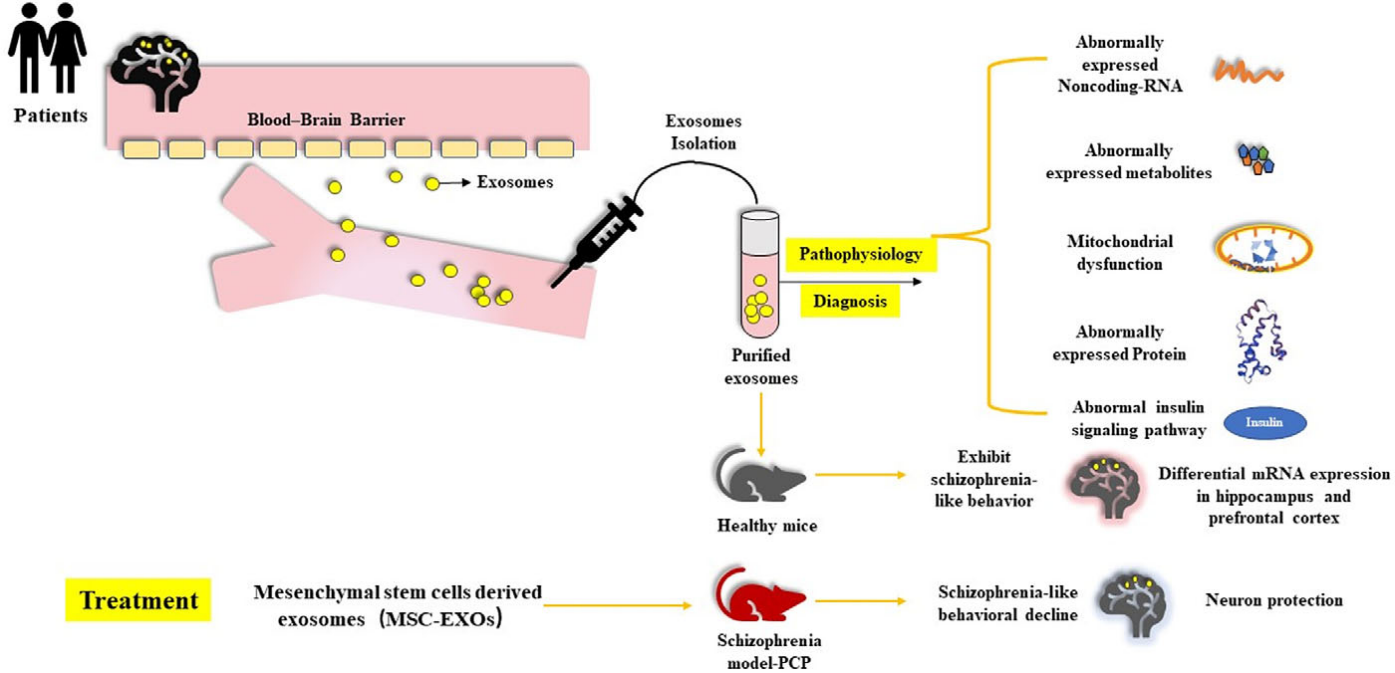
Exosome in schizophrenia: pathophysiology, diagnosis and treatment.

Phencyclidine (PCP) is an N-methyl-D-aspartate (NMDA-R) receptor antagonist that induces neurocognitive impairments mimicking SCZ via the inhibition of glutamate transmission [90]. As MSCs-derived EVs have shown to concentrate on the lesion in the brain [32], Tsivion-Visbord et al. [91] allocated mice into three groups, namely EVs + PCP treated group, PCP-treated group, and saline group at random, and compared behavioral changes after intranasal administration of exosomes derived from MSCs to the three groups. Their results revealed that treatment with MSC-exos ameliorated SCZ-like behaviors, including deficits in social interaction and prepulse inhibition. The authors also observed an accumulation of MSC-exos along with the preservation of PV-positive GABAergic interneurons in the prefrontal cortex. The aggregation of MSC-exos in the prefrontal cortex potentially indicates its vulnerability to damage caused PCP and hence, pointing at its significant implication in SCZ and SCZ-like symptoms produced by PCP. Furthermore, the preservation of PV-positive GABAergic interneurons restored glutamate levels in the cerebrospinal fluid which improved the SCZ-like behaviors of the PCP-treated mice administered with MSC-exos [91]. Overall, these studies imply that exosomes can potentially be employed to develop/transport drugs and thus improve the treatment of SCZ (Figure 2).

## Conclusion and Outlook

The development of experimental techniques, such as qRT-PCR and high-throughput sequencing, has promoted the study of exosomes and their functions. Multiple lines of evidence have suggested the potential involvement of exosomes in the occurrence and development of SCZ. In addition, it has been reported that exosomal contents, such as miRNA, circular RNA, metabolites, and so forth, can serve as reliable biomarkers for the diagnosis of SCZ. Therefore, exosomes can specifically be used for the early detection of SCZ. Furthermore, they can efficiently deliver therapeutic cargo for treatment by carrying specific small RNAs. In particular, MSC-exos, which are directly transported to damaged brain tissues, can alleviate the behavioral symptoms of SCZ and are thus a favorable method to treat neuropsychiatric disorders in a targeted way. Nevertheless, it is not yet known whether the alterations in the diversity and composition of exosomes are the cause or outcome of SCZ.

There are a few limitations that should be taken into account when investigating the use exosomes in SCZ. Firstly, there exist numerous ways to isolate and purify exosomes, the markers currently used to define exosomes overlap largely with other classes of EV such as microvesicles, and thus the results obtained by different methods might be inconsistent. Secondly, metabolomics research technology is not yet mature, and their results are not necessarily accurate. Thirdly, the mechanism of differentially expressed contents in exosomes, how they mediate the occurrence and development of diseases and even the mechanism of exosome treatment still remains unclear. Also, the symptoms exhibited by mouse models are not specific and there is heterogeneity between different animal models of SCZ or between human and animal models. Therefore, it is difficult to translate the results of mice models directly to humans and more research is required so as to further determine the mechanism and safety of exosomes in the treatment of diseases. Lastly, high-throughput proteomics analysis of exosomes has been widely used in the study of neurodegenerative diseases but not in SCZ.

In summary, while exosomes are promising potential markers for the diagnosis and treatment of SCZ, the next step is to study their contents in addition to their involvement in SCZ in a multi-omics and longitudinal manner which will not only allow a better comprehension of the pathogenesis of SCZ but will also allow us to employ exosomes in the treatment SCZ at a molecular level in the future.
